# ZnPc(Lys)_5_-mediated photodynamic inactivation and reduction of *Candida* albicans biofilm-associated cells *in vitro* and *in vivo*

**DOI:** 10.3389/fbioe.2026.1953510

**Published:** 2026-10-07

**Authors:** Danlin Li, Fang Yuan, Yuchen Yang, Kangli Zhou, Fei Xie, Zhiming Li, Xiaoling Huang, Mingdong Huang, Minkui Lin

**Affiliations:** 1 Clinical Research Center for Oral Tissue Deficiency Diseases of Fujian Province and Fujian Key Laboratory of Oral Diseases and Fujian Provincial Engineering Research Center of Oral Biomaterial, School and Hospital of Stomatology, Fujian Medical University, Fuzhou, China; 2 Institute of Stomatology and Laboratory of Oral Tissue Engineering, School and Hospital of Stomatology, Fujian Medical University, Fujian, China; 3 Department of Stomatology, Cangshan Hospital, the 900Th Hospital of Joint Logistics Support Force of Chinese People’s Liberation Army, Fuzhou, China; 4 College of Chemistry, Fuzhou University, Fuzhou, China

**Keywords:** animal model of candidal stomatitis, antimicrobial photodynamic therapy (aPDT), *Candida* albicans, denture stomatitis, polymethyl methacrylate (PMMA)

## Abstract

**Introduction:**

Candida-associated stomatitis is a common oral mucosal condition, particularly among denture wearers, and is closely associated with persistent *Candida* albicans biofilms on denture surfaces. ZnPc(Lys)_5_ is an amphiphilic phthalocyanine–peptide nanomaterial that has previously been shown to form nanoscale supramolecular assemblies in aqueous environments. This study evaluated the efficacy of ZnPc(Lys)_5_-mediated antimicrobial photodynamic therapy (aPDT) in reducing denture-associated *Candida* biofilms and alleviating associated mucosal inflammation.

**Methods:**

*Candida* albicans suspensions were cultured *in vitro*, and biofilms were formed on polymethyl methacrylate (PMMA), a common denture base material. The antifungal effects of different concentrations of ZnPc(Lys)_5_ combined with aPDT were assessed against both planktonic *Candida* and biofilms. Biofilm structural changes were observed by scanning electron microscopy, and the effects on PMMA physical properties (microhardness [HV] and surface roughness [Ra]) were analyzed. Additionally, a mouse model of Candida-associated stomatitis was used to evaluate the *in vivo* anti-inflammatory and antifungal efficacy of ZnPc(Lys)_5_-aPDT following repeated applications (three treatments over 5 days).

**Results:**

ZnPc(Lys)_5_-mediated aPDT exerted a significant concentration-dependent fungicidal effect on planktonic *C. albicans*, with a minimum effective concentration (MEC) of 25 μM and no observed dark toxicity. Against mature biofilms, the 50 μM aPDT treatment achieved a reduction of approximately 96% in recoverable viable biofilm-associated cells (P < 0.0001), which was comparable to the efficacy of the commercial cleanser Polident (P > 0.05). Scanning electron microscopy confirmed significant disruption of biofilm architecture, while no significant alterations in PMMA microhardness or surface roughness were observed (P > 0.05). *In vivo*, repeated applications of ZnPc(Lys)_5_-aPDT significantly reduced epithelial hyperplasia and inflammatory infiltration, demonstrating therapeutic efficacy comparable to nystatin.

**Conclusion:**

ZnPc(Lys)_5_-aPDT demonstrated significant antifungal activity *in vitro* and *in vivo* without compromising PMMA integrity. These findings support ZnPc(Lys)_5_ as a promising photosensitizer for denture-surface biofilm control and mucosal inflammation mitigation, warranting further investigation under extended or multi-session clinical protocols.

## 1 Introduction

Denture stomatitis (DS) is a common chronic inflammatory condition of the oral mucosa that predominantly affects denture wearers and is frequently associated with oral candidiasis (Gendreau and Loewy, 2011; Iacopino and Wathen, 1992). Clinically, DS presents as erythema and edema of the mucosa beneath removable dental prostheses and may be accompanied by discomfort or burning sensations (Gendreau and Loewy, 2011; Alves et al., 2020). DS is now recognized as a multifactorial condition involving microbial biofilms, inadequate denture hygiene, mechanical irritation, salivary alterations, systemic conditions, and host immune responses (Abuhajar et al., 2023; McReynolds et al., 2023; Le Bars et al., 2022; P et al., 2024; Budtz-Jorgensen, 2000). Among these contributing factors, denture-surface colonization by *Candida* albicans and the subsequent formation of structured biofilms are considered major microbial contributors to DS (P et al., 2024; Budtz-Jorgensen, 2000). Although *C. albicans* is a common opportunistic commensal of the oral cavity (Schille et al., 2025; Li et al., 2022), microbial dysbiosis or impaired host immunity can promote its transition to a pathogenic state. Fungal cells subsequently adhere to denture base materials and form mature biofilms that act as persistent reservoirs for mucosal recolonization, thereby sustaining local inflammation and contributing to disease recurrence (Ramage et al., 2025). Within these biofilms, *C. albicans* cells are embedded in an extracellular polymeric substance (EPS) matrix that restricts antifungal penetration, protects fungal cells from host immune defenses, and promotes long-term persistence (Roy and Gow, 2023; Atriwal et al., 2021; Fan et al., 2022). These features render mature biofilms substantially more difficult to eliminate than planktonic cells and underscore the importance of effective biofilm control, particularly in elderly and immunocompromised individuals.

Polymethyl methacrylate (PMMA) remains the most widely used denture base material because of its favorable mechanical properties and biocompatibility (de Oliveira Limírio et al., 2022). However, surface irregularities, residual porosity, and hydrophobic interactions associated with conventionally processed PMMA can facilitate microbial adhesion and biofilm development, creating a favorable niche for *C. albicans* colonization (Keleştemur et al., 2020). The combination of PMMA surface characteristics and the structural resilience of fungal biofilms makes effective denture decontamination challenging in clinical settings (Paranhos et al., 2007; Kreve and Reis, 2025). Current control strategies have several limitations. Mechanical cleaning may fail to disrupt mature biofilms or remove microorganisms retained within PMMA microstructures (Paranhos et al., 2007). Chemical denture cleansers can reduce microbial contamination, but their efficacy varies according to formulation, exposure time, and biofilm maturity, while repeated or prolonged use may adversely affect certain denture material properties (Peracini et al., 2010; Ozyurt Tekin and Caglar, 2024). Conventional antifungal agents, including nystatin and azoles, are generally less effective against established *C. albicans* biofilms than against planktonic cells (Silva et al., 2017). Moreover, prolonged or repeated antifungal therapy may be associated with reduced susceptibility, recurrence, and poor patient adherence (He et al., 2020; Bhattacharya et al., 2020). These limitations highlight the need for alternative approaches capable of controlling denture-associated *Candida* biofilms without compromising denture material integrity.

Antimicrobial photodynamic therapy (aPDT) has emerged as a promising approach for biofilm control (Hu et al., 2018; Baltazar et al., 2015; Li et al., 2023). In aPDT, a photosensitizer is activated by light of an appropriate wavelength in the presence of oxygen, generating reactive oxygen species (ROS) that induce oxidative damage at multiple microbial cellular targets (Dias et al., 2023; Gonzales and Maisch, 2012; Carvalho et al., 2018). Among these species, singlet oxygen has a lifetime in the nanosecond range and a diffusion distance generally limited to the nanometer scale, making photosensitizer localization critical to the spatial distribution of photodynamic damage. Because aPDT acts through oxidative injury at multiple targets, it is considered to have a relatively low propensity for inducing microbial resistance and has demonstrated activity against biofilm-associated microorganisms (Liu et al., 2015; Tanu et al., 2025; Dai et al., 2012). When photosensitizers are effectively associated with fungal cells or extracellular biofilm components, locally generated ROS can induce oxidative damage within the biofilm microenvironment and inactivate microorganisms that exhibit reduced susceptibility to conventional treatments (Maisch et al., 2007; Mang et al., 2016; Tonon et al., 2021; Misba et al., 2018). However, aPDT efficacy depends strongly on photosensitizer properties, including aqueous solubility, aggregation behavior, microbial association, light absorption, and ROS generation. Some conventional photosensitizers show limited activity against mature fungal biofilms because of inadequate biofilm accessibility, aggregation-induced photoinactivation, or insufficient retention at microbial interfaces (Bliss et al., 2004; Kwi et al., 2018; Costa et al., 2012; da Silva et al., 2017). Molecular modification of phthalocyanines with hydrophilic or cationic functional groups has therefore been explored to improve their aqueous compatibility, microbial interactions, and photodynamic performance.

ZnPc(Lys)_5_ is an amphiphilic phthalocyanine–peptide conjugate that spontaneously self-assembles into spherical supramolecular nanodots in aqueous media, with the hydrophobic phthalocyanine cores stacked internally and the positively charged pentalysine chains forming a hydrophilic shell. In aqueous solution, this nano-assembly state is spectrally characterized by a dominant aggregate absorption peak around 630 nm (H-type stacking) with quenched fluorescence emission. These nano-assemblies exist in dynamic equilibrium with photoactive monomers, and interactions with cellular or bacterial membranes can promote aggregate disassembly and monomer release (Chen et al., 2024; Jia et al., 2018; Liu et al., 2023). ZnPc(Lys)_5_-mediated aPDT has demonstrated antibacterial activity against a range of bacterial pathogens, including multidrug-resistant strains, at relatively low photosensitizer concentrations and irradiation doses, with therapeutic efficacy further demonstrated in localized infected-wound models (Chen et al., 2024; Ullah et al., 2018; Wang et al., 2017). However, its antifungal efficacy against *C. albicans*, particularly against denture-associated biofilms and associated mucosal inflammation, remains insufficiently investigated. To our knowledge, limited information is currently available regarding the antifungal and antibiofilm effects of ZnPc(Lys)_5_-mediated aPDT against C. albicans planktonic cells and PMMA-associated biofilms. Therefore, the present study provides a comprehensive evaluation of its efficacy, determines the minimum effective concentration against planktonic cells, and assesses its therapeutic potential in a mouse model of Candida-associated stomatitis, thereby providing a potential strategy for controlling denture-associated *Candida* biofilms and related mucosal inflammation.

## 2 Materials and methods

### 2.1 Photosensitizer and light source

ZnPc(Lys)_5_ was synthesized and provided by the Mingdong Huang research group at Fuzhou University (Fuzhou, China) and has the chemical formula C_63_H_76_N_18_O_7_Zn (Chen et al., 2024). ZnPc(Lys)_5_ was dissolved in pure water, prepared as a 5 mM stock solution, and stored in the dark at −80 °C. Before use, the stock solution was diluted with phosphate-buffered saline (PBS) to the desired concentration. All working solutions were freshly prepared prior to each experiment. ZnPc(Lys)_5_ exhibited a typical UV/visible absorption spectrum in dimethyl sulfoxide (DMSO), with the strongest absorption peak at 678 nm (ε = 118,380 L/mol·cm).

A light-emitting diode (LED) light source (China Nanning Sanyi Lighting Co., Ltd.; wavelength 680 nm; output power density 25 mW/cm^2^) was used for irradiation. The irradiation dose applied in all photodynamic therapy experiments was 18 J/cm^2^. The irradiation time (12 min) was calculated based on the measured power density to achieve the intended energy dose (Energy density = power density × time).

### 2.2 *Candida albicans* strain and culture conditions

The *C. albicans* standard strain (BNCC299343) was provided by the Ming Zheng research group (School and Hospital of Stomatology, Fujian Medical University). The strain was cultured aerobically on Sabouraud dextrose agar (SDA) plates at 37 °C.

For fungicidal assays using planktonic suspensions and for *in vivo* induction of candidal stomatitis, fungal suspensions were prepared using PBS buffer. For biofilm formation experiments, yeast extract peptone dextrose (YPD) medium was used.

### 2.3 Sample preparation

The denture base polymethyl methacrylate (PMMA) resin powder and liquid (Shanghai New Century Dental Materials Co., Ltd., China) were mixed according to the manufacturer’s instructions to obtain a homogeneous mixture. The mixture was injected into molds at the dough stage and polymerized using pressurized low-temperature curing (55 °C ± 5 °C for 30 min). After curing, specimens were allowed to cool naturally.

All specimens were sequentially polished in the same direction for 30 s using 600-, 800-, and 1000-grit waterproof silicon carbide sandpapers to obtain a standardized surface finish. After polishing, surface roughness (Ra) was measured using a profilometer. The thickness, length, and width of each specimen were measured using an electronic digital caliper with an accuracy of ±0.02 mm, and all specimens were standardized to dimensions of 10.0 mm × 10.0 mm × 2.0 mm. Specimens were ultrasonically cleaned for 30 min and stored in sterile PBS at 37 °C for 52 ± 2 h to allow residual monomer release. Subsequently, specimens were air-dried at room temperature, immersed in 75% ethanol for 30 min, and sterilized by ultraviolet irradiation for 30 min prior to use.

Each experimental group consisted of five replicate specimens per independent experiment. For all *in vitro* quantitative assays, each experiment was independently repeated three times on different days using freshly prepared specimens and reagents (biological replicates, N = 3). Within each biological replicate, the five replicate specimens served as technical replicates (n = 5). To avoid pseudoreplication, the mean value of the five technical replicates was calculated for each biological replicate, and this mean was used as a single data point for statistical analysis. Thus, the sample size for inferential statistics was N = 3 independent biological experiments. For the planktonic cell assay (Section 2.4; Figure 1), the experimental design was similar but with four independent biological replicates (N = 4), each comprising three technical replicate plates (n = 3).

**FIGURE 1:**
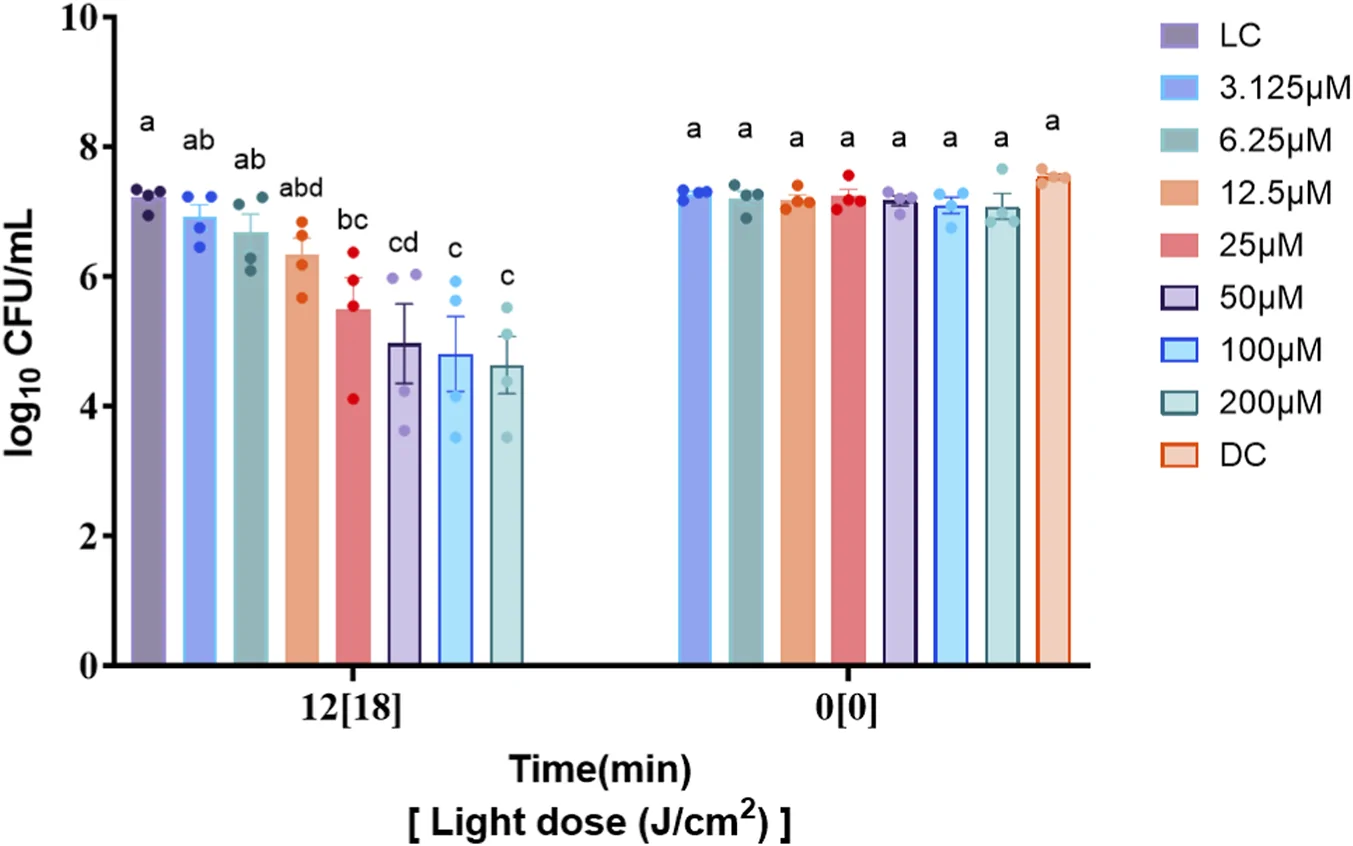
Comparative analysis of the *in vitro* sterilization effects of *C. albicans* suspensions with different photosensitizer concentrations. Data are presented as mean ± SD, n = 4 per group. One-way ANOVA followed by Tukey’s HSD multiple comparisons test was performed on all sixteen groups (eight light-treated and eight dark-treated groups). Different lowercase letters above the bars indicate significant differences between groups (P < 0.05); groups sharing the same letter are not significantly different. Exact adjusted P-values, mean differences (effect sizes), and 95% confidence intervals are provided in Supplementary Table S1.

### 2.4 *In vitro* photodynamic therapy against *Candida albicans* suspension

*C. albicans* was cultured on SDA plates at 37 °C for 48 h. A single isolated colony was inoculated into 10 mL of YPD liquid medium and incubated in a constant-temperature shaker at 28 °C and 200 rpm for 16 h. The culture was centrifuged at 5,000 rpm at 4 °C for 5 min, washed three times with PBS, and resuspended in YPD medium.

The fungal suspension was adjusted to a concentration of 4.0–5.0 × 10^7^ colony-forming units (CFU)/mL, equivalent to a 0.5 McFarland standard. Cell density was verified by measuring OD600 with a UV–visible spectrophotometer using sterile YPD medium as a blank; based on a previously established standard growth curve in our laboratory, an OD600 of approximately 1.0 corresponds to 4.0–5.0 × 10^7^ CFU/mL. The OD600 values used in this study ranged from 0.86 to 1.06.

For the antimicrobial photodynamic therapy (aPDT) group, 50 μL of fungal suspension was added to each well of a 96-well plate, followed by the addition of 50 μL of ZnPc(Lys)_5_ at different concentrations to achieve final concentrations ranging from 3.125 to 200 μM (total volume: 100 μL). After incubation in the dark at 37 °C for 10 min, samples were irradiated with LED light at a dose of 18 J/cm^2^.

The dark toxicity control group received the same treatment without light irradiation. The phototoxicity control group received fungal suspension and PBS with light irradiation only. The negative control group received fungal suspension and PBS without photosensitizer or light exposure.

After treatment, 20 μL of each suspension was serially diluted and plated on SDA plates. Colonies were counted after incubation at 37 °C for 48 h to assess antifungal efficacy. For this assay, each experimental condition was tested in triplicate technical replicates within each of four independent biological experiments (N = 4), with the mean of the three technical replicates used as a single data point for statistical analysis.

### 2.5 *In vitro* photodynamic therapy against PMMA surface biofilms

Sterile PMMA specimens were placed in 24-well plates. A fresh *C. albicans* suspension was prepared as described above and adjusted to 1 × 10^8^ CFU/mL (OD_600_ = 2.06). A volume of 100 μL of the suspension was inoculated onto each PMMA surface and incubated at 37 °C for 90 min to allow initial adhesion. Subsequently, 900 μL of YPD medium was added, and samples were incubated at 37 °C for 48 h to allow biofilm formation, with fresh medium replaced every 24 h.

After biofilm formation, specimens were gently washed twice with PBS to remove non-adherent cells. For the aPDT group, specimens were immersed in 1 mL of ZnPc(Lys)_5_ solution (12.5, 25, or 50 μM) and incubated in the dark at room temperature for 10 min, followed by LED irradiation at 18 J/cm^2^.

The negative control group was treated with sterile PBS. The positive control group was immersed in Polident cleaning solution for 5 min, which was included as a representative commercial denture cleanser commonly used in clinical practice rather than as a pharmacological antifungal agent. In addition, a light-only control group (LED irradiation at 18 J/cm^2^ without ZnPc(Lys)_5_) and three ZnPc(Lys)_5_-only (dark) control groups (12.5, 25, and 50 μM, without light irradiation) were included to verify that neither light exposure nor the photosensitizer alone exerted antifungal effects.

Notably, the treatment durations differed between modalities because each followed its respective standard clinical or experimental protocol: Polident immersion for 5 min follows the manufacturer’s instructions and prosthodontic cleaning guidelines, whereas aPDT required a 10-min dark incubation to allow photosensitizer penetration into the biofilm and disassembly of nanoaggregates into photoactive monomers, followed by 12 min of LED irradiation at 25 mW/cm^2^ to deliver an effective light dose of 18 J/cm^2^. Accordingly, this comparison represents an endpoint efficacy evaluation under each modality’s standard operating conditions rather than a time-normalized kinetic comparison.

After treatment, specimens were rinsed with PBS, placed in centrifuge tubes containing 2 mL PBS, and vortexed three times for 2 min each to detach biofilm cells. Aliquots (20 μL) of the resulting suspensions were plated on SDA and incubated at 37 °C for 48 h for colony counting.

The biofilm CFU assay was re-designed with nine complete groups to present a full set of concentration-matched controls: (1) PBS negative control, (2) light-only control (LED 18 J/cm^2^), (3–5) ZnPc(Lys)_5_-only (dark) controls at 12.5, 25, and 50 μM, (6–8) ZnPc(Lys)_5_-aPDT groups at 12.5, 25, and 50 μM (18 J/cm^2^), and (9) Polident positive control. This expanded design was adopted because, although the original four-group design yielded a large effect size (η^2^ = 0.518), the limited sample size constrained statistical power. The complete descriptive statistics, ANOVA outputs, and all pairwise comparison results are provided in Supplementary Table S2.

### 2.6 Scanning electron microscopy (SEM)

PMMA specimens with preformed biofilms were divided into five groups (n = 5 per group): untreated control, Polident-treated group, ZnPc(Lys)_5_ dark group (50 μM, 10 min), light-only group (18 J/cm^2^), and aPDT group (50 μM ZnPc(Lys)_5_ followed by LED irradiation).

After treatment, specimens were fixed in 2.5% glutaraldehyde at 4 °C, dehydrated through a graded ethanol series (50%–100%), sputter-coated with gold, and observed using a scanning electron microscope (Quanta 250, Thermo Fisher Scientific, United States) at an accelerating voltage of 15 kV. SEM was used for qualitative assessment of biofilm morphology; quantitative biofilm biomass analyses were not performed in the present study.

### 2.7 Physical property testing of PMMA

PMMA specimens were divided into five treatment groups (n = 5 technical replicates per biological experiment): (1) 50 μM ZnPc(Lys)_5_ dark-only group, (2) no decontamination (untreated) control group, (3) 50 μM ZnPc(Lys)_5_-PDT group (50 μM ZnPc(Lys)_5_ followed by LED irradiation at 18 J/cm^2^), (4) LED-only group (18 J/cm^2^ without ZnPc(Lys)_5_), and (5) Polident positive control group. Each specimen was measured three times by the instrument, and the mean of the three readings was used as the representative value for that specimen. The mean of the five specimen values was then calculated for each independent biological experiment (N = 3), and this mean served as a single data point for statistical analysis. Surface roughness (Ra) was measured using a profilometer (DSF600, Kosaka Laboratory, Japan) with a resolution of 0.01 μm, a measurement interval of 0.8 mm, a lateral length of 2.0 mm, and a stylus speed of 0.5 mm/s.

Microhardness (HV) was measured using a Vickers hardness tester (HX-1000TM, Shanghai Taiming Optical Instrument Co., Ltd., China) with a 300 g load applied for 30 s. All measurements were performed by a single calibrated examiner who was blinded to the group allocation. Each specimen was measured three times, and the mean value was recorded.

### 2.8 Experimental animals

Adult male ICR mice (6–8 weeks old, ∼30 g) were obtained from Beijing Huafu Kangsheng Biotechnology Co., Ltd.

Animal experiments were approved by the Biomedical Ethics Committee of the Fujian Provincial Center for Disease Control and Prevention (No. PZ2025002).

All procedures were conducted in accordance with institutional guidelines for animal care and complied with the ARRIVE guidelines. Mice were housed under specific pathogen-free conditions with controlled temperature (22 °C ± 2 °C), humidity (50%–60%), and a 12 h light/dark cycle, with free access to food and water.

### 2.9 Mouse *Candida albicans* stomatitis infection model

The *C. albicans* infection model was established on the basis of a previously published study (Solis and Filler, 2012; Costa et al., 2013). One day prior to infection, mice received a subcutaneous injection of cortisone acetate (225 mg/kg) to induce immunosuppression.

Before sublingual inoculation, mice were anesthetized by intraperitoneal injection of 1.25% tribromoethanol at 20 mL/kg. After anesthesia, the animals were kept warm and monitored throughout the procedure and subsequent recovery.

*C. albicans* suspensions were prepared at 3 × 10^6^ CFU/mL, and sublingual inoculation was performed using calcium alginate swabs soaked in 0.2 mL of suspension and applied for 75 min. Additional cortisone acetate injections (225 mg/kg) were administered on days 1, 3, and 5 post-inoculation. Successful establishment of infection was confirmed 5 days after inoculation by the presence of visible pseudomembranous lesions on the dorsal tongue surface. In addition, PAS staining was performed in pilot animals prior to intervention to verify fungal colonization of the superficial epithelium.

### 2.10 *In vivo* aPDT intervention

Eighteen mice were randomly assigned to three experimental groups (n = 6 per group): (1) stomatitis-negative control (infected + PBS), (2) aPDT group (infected +50 μM ZnPc(Lys)_5_ followed by LED irradiation), and (3) nystatin treatment group (infected + nystatin). In addition, a separate cohort of uninfected mice (n = 3) was included as a normal histological reference to establish the baseline tongue epithelial architecture. Group allocation was performed using a random number table.

Before each treatment session, mice were anesthetized by intraperitoneal injection of 1.25% tribromoethanol at 20 mL/kg, as described above. In the aPDT group, 50 μL of ZnPc(Lys)_5_ solution (50 μM) was applied to the dorsal tongue surface and incubated for 10 min, followed by LED irradiation at 25 mW/cm^2^ for 12 min (18 J/cm^2^). Treatments were administered once every other day, for a total of three treatments within 5 days.

Nystatin was applied once every other day (three applications within 5 days), with the drug maintained in contact with the dorsal tongue surface for 10 min per treatment, following the same treatment schedule as the aPDT group.

The negative control group received PBS following the same schedule. All mice were euthanized 24 h after the final treatment by gradual-fill CO_2_ inhalation. The mice were initially exposed to a low CO_2_ concentration of 10%–30%, after which CO_2_ was introduced at a displacement rate of approximately 20% of the chamber volume per minute until a concentration of at least 70% was reached. This concentration was maintained for 5 min. Death was confirmed by cardiac arrest and fixed pupillary dilation before tissue collection. Tongue tissues were harvested, fixed in 4% paraformaldehyde, dehydrated, paraffin-embedded, and sectioned at 5 μm thickness for histological analysis.

### 2.11 Histopathological examination

Hematoxylin and eosin (H&E) staining and periodic acid–Schiff (PAS) staining were performed following standard protocols. Sections were examined under an inverted microscope (CKX41, Olympus Corporation, Japan) and photographed. Histopathological evaluations were conducted by an investigator blinded to treatment groups.

### 2.12 Statistical analysis

All quantitative experiments were independently repeated as follows: the planktonic cell assay (Figure 1) was performed in four independent biological experiments (N = 4), while the biofilm reduction assay (Figure 2), SEM, and PMMA physical property tests (Figure 3) were each performed in three independent biological experiments (N = 3). Within each biological replicate, technical replicates were included as described in the respective methods sections. For all quantitative analyses, the mean of technical replicates was calculated to yield a single data point per biological replicate, thereby avoiding pseudoreplication.

**FIGURE 2:**
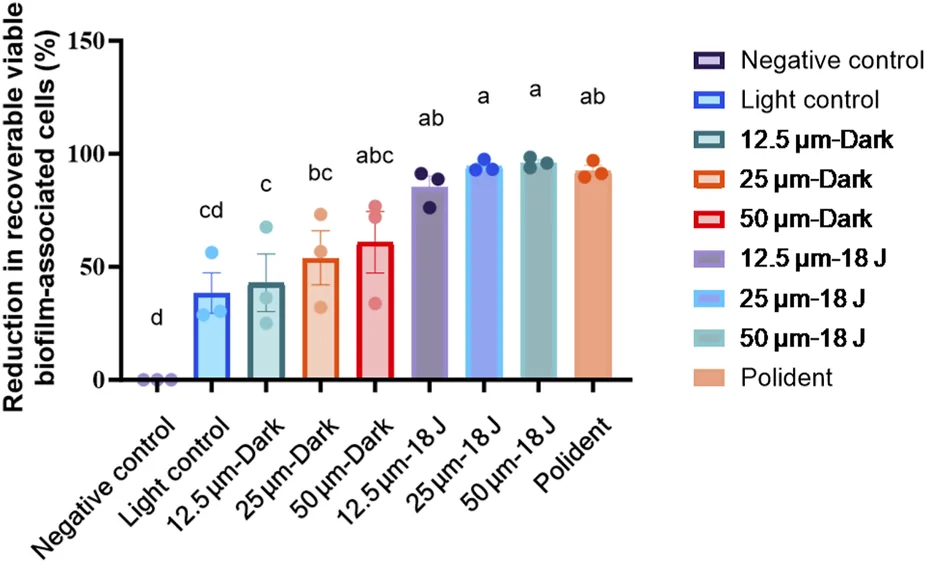
Comparative analysis of the *in vitro* antibiofilm effects of ZnPc(Lys)_5_-mediated photodynamic therapy on *C. albicans* biofilms. Biofilm-coated PMMA specimens were treated with ZnPc(Lys)_5_ at different concentrations (12.5, 25, and 50 μM) combined with light irradiation (18 J/cm^2^). The reduction in recoverable viable biofilm-associated cells is expressed as a percentage (%) relative to the negative control. Data are presented as mean ± SD (n = 3 per group). Statistical significance was determined by one-way ANOVA followed by Tukey’s HSD multiple comparisons test. Different lowercase letters above the bars indicate significant differences between groups (P < 0.05); groups sharing the same letter are not significantly different. Abbreviations: DC, dark/negative control (no light, no photosensitizer); LC, light control (light only, no photosensitizer); Polident, positive control. Exact adjusted P-values, mean differences, and 95% confidence intervals are provided in Supplementary Table S2.

**FIGURE 3:**
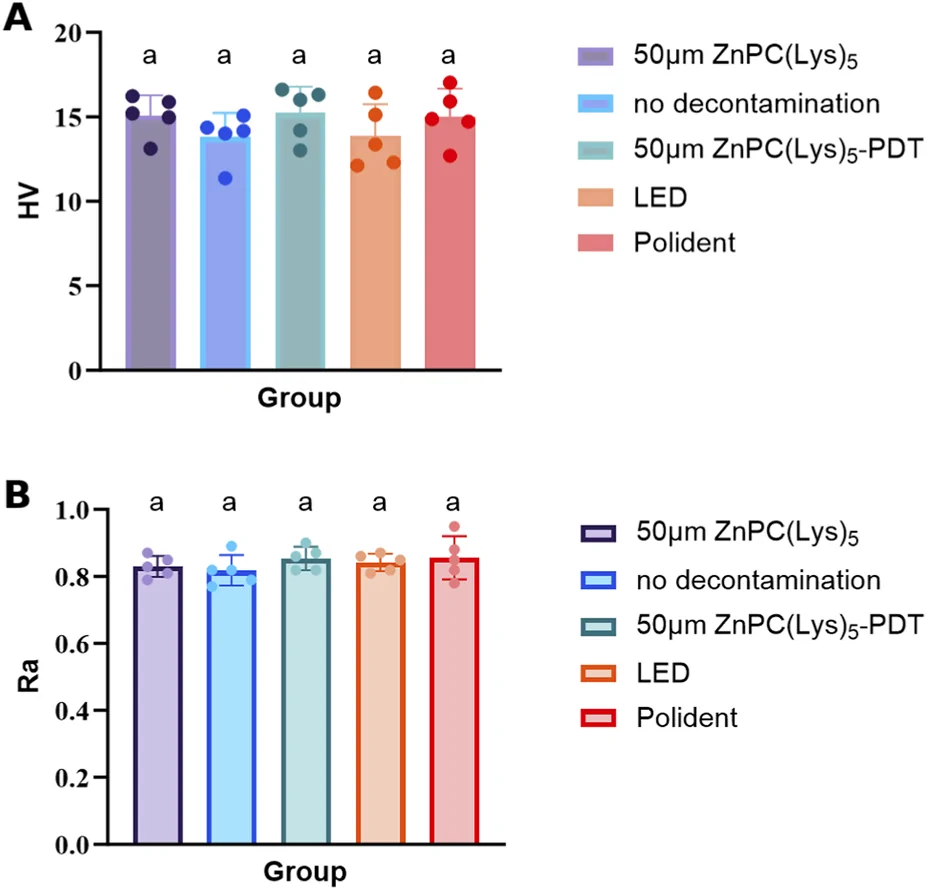
Physical properties of the PMMA samples following different treatment schemes. **(A)** Comparison of Vickers hardness values among the five groups. Bars represent mean values, dots represent individual replicates. Groups sharing the same lowercase letter (a) are not significantly different (Tukey’s multiple comparisons test, P > 0.05). Full pairwise comparison results are provided in Supplementary Table S3. **(B)** Comparison of surface roughness values among the five groups. Bars represent mean values, dots represent individual replicates. Groups sharing the same lowercase letter (a) are not significantly different (Tukey’s multiple comparisons test, P > 0.05). Full pairwise comparison results are provided in Supplementary Table S4.

Data are expressed as mean ± standard deviation (SD). One-way analysis of variance (ANOVA) was performed for all quantitative datasets. For the planktonic cell assay (Figure 1; Supplementary Table S1) and the biofilm reduction assay (Figure 2; Supplementary Table S2), ANOVA was followed by Tukey’s *post hoc* test for multiple comparisons. For PMMA physical property assessments (Figure 3; Supplementary Tables S3, S4), ANOVA was also followed by Tukey’s *post hoc* test for multiple comparisons. For ANOVA, the F-statistic, degrees of freedom (df), and effect size (partial η^2^) are reported. For pairwise comparisons, mean differences with 95% confidence intervals are reported. Exact P values (to four decimal places) are provided throughout the Results and Figure Legends; P < 0.0001 is used when the value is less than 0.0001. All statistical analyses were performed using GraphPad Prism 10. H&E and PAS staining results were assessed qualitatively.

## 3 Results

### 3.1 Antimicrobial photodynamic effects of ZnPc(Lys)_5_ on *Candida albicans* suspension

The photodynamic inactivation (PDI) efficacy of ZnPc(Lys)_5_ against planktonic *C. albicans* was evaluated. One-way ANOVA revealed a statistically significant difference among the groups (F (15, 48) = 11.13, P < 0.0001). Post-hoc Tukey HSD tests, using the untreated control (DC, mean 7.54 log_10_ CFU/mL) as a reference, showed that in the irradiated groups, concentrations of 3.125–12.5 μM did not cause a statistically significant reduction in colony counts (P > 0.05). However, a concentration of 25 μM significantly reduced the viable cell count to 5.49 log_10_ CFU/mL (mean difference −2.05, P = 0.0013). The fungicidal effect reached a plateau at 50, 100, and 200 μM, with reductions of 2.57, 2.75, and 2.90 log_10_, respectively, all of which were highly significant compared to the DC group (P < 0.0001). Pairwise comparisons among the 25 μM–200 μM groups showed no significant differences (P > 0.05). In the non-irradiated groups, no significant differences in cell viability were observed for any tested concentration (3.125–200 μM) compared to the DC group (P ≈ 1.000). Detailed results of all pairwise comparisons are presented in Supplementary Table S1.

### 3.2 ZnPc(Lys)_5_-mediated photodynamic reduction of preformed biofilms on PMMA surfaces

Given the potent antifungal activity observed in planktonic cells, we next evaluated the ability of ZnPc(Lys)_5_-mediated aPDT to reduce fungal recovery from preformed *C. albicans* biofilms on PMMA surfaces. Biofilm-coated PMMA specimens were immersed in ZnPc(Lys)_5_ solutions (12.5–50 μM) for 10 min followed by aPDT treatment (18 J/cm^2^). The experiment comprised nine groups: PBS negative control, light-only control (LED 18 J/cm^2^), ZnPc(Lys)_5_-only (dark) controls at 12.5, 25, and 50 μM, ZnPc(Lys)_5_-aPDT groups at 12.5, 25, and 50 μM (18 J/cm^2^), and Polident positive control.

ZnPc(Lys)_5_-mediated aPDT resulted in a concentration-dependent trend of biofilm viability reduction. As shown in Figure 2, the log_10_ reductions in recoverable CFUs were 0.84 ± 0.19 (12.5 μM), 1.28 ± 0.11 (25 μM), and 1.41 ± 0.09 (50 μM), corresponding to percentage reductions of 85.45%, 94.53%, and 96.08%, respectively, whereas the Polident group showed a log_10_ reduction of 1.14 ± 0.16 (92.66%). Statistical analysis indicated no significant difference among the three aPDT concentrations and the Polident positive control (12.5 μM and Polident labeled “ab”; 25 and 50 μM labeled “a”; all sharing the letter “a”), although the antibiofilm effect appeared to approach a plateau at 25 μM. Notably, the 50 μM aPDT group achieved a reduction comparable to that of the Polident positive control, demonstrating equivalent efficacy to the commercial cleanser.

Regarding safety and specificity, the light-only control and the 12.5 and 25 μM dark toxicity controls showed minimal reduction in cell viability compared with the negative control (labeled “d” and “cd”, respectively), indicating low inherent toxicity. Although the 50 μM dark control exhibited a moderate reduction (approx. 60%, labeled “abc”), all aPDT groups exhibited significantly greater reductions than their concentration-matched dark controls (P < 0.05). This confirms that the primary biofilm inactivation was attributable to the photodynamic reaction rather than to light exposure or dark toxicity alone. Full descriptive statistics and exact adjusted P-values are provided in Supplementary Table S2.

### 3.3 Scanning electron microscopy (SEM) analysis

SEM images (Figure 4) revealed dense and well-organized *C. albicans* biofilms on the PMMA surface in the blank control group (Figure 4A). In the illumination-only control group (Figure 4B), no apparent reduction in fungal density was observed compared with the blank control, suggesting that light exposure alone did not substantially affect biofilm integrity.

**FIGURE 4:**
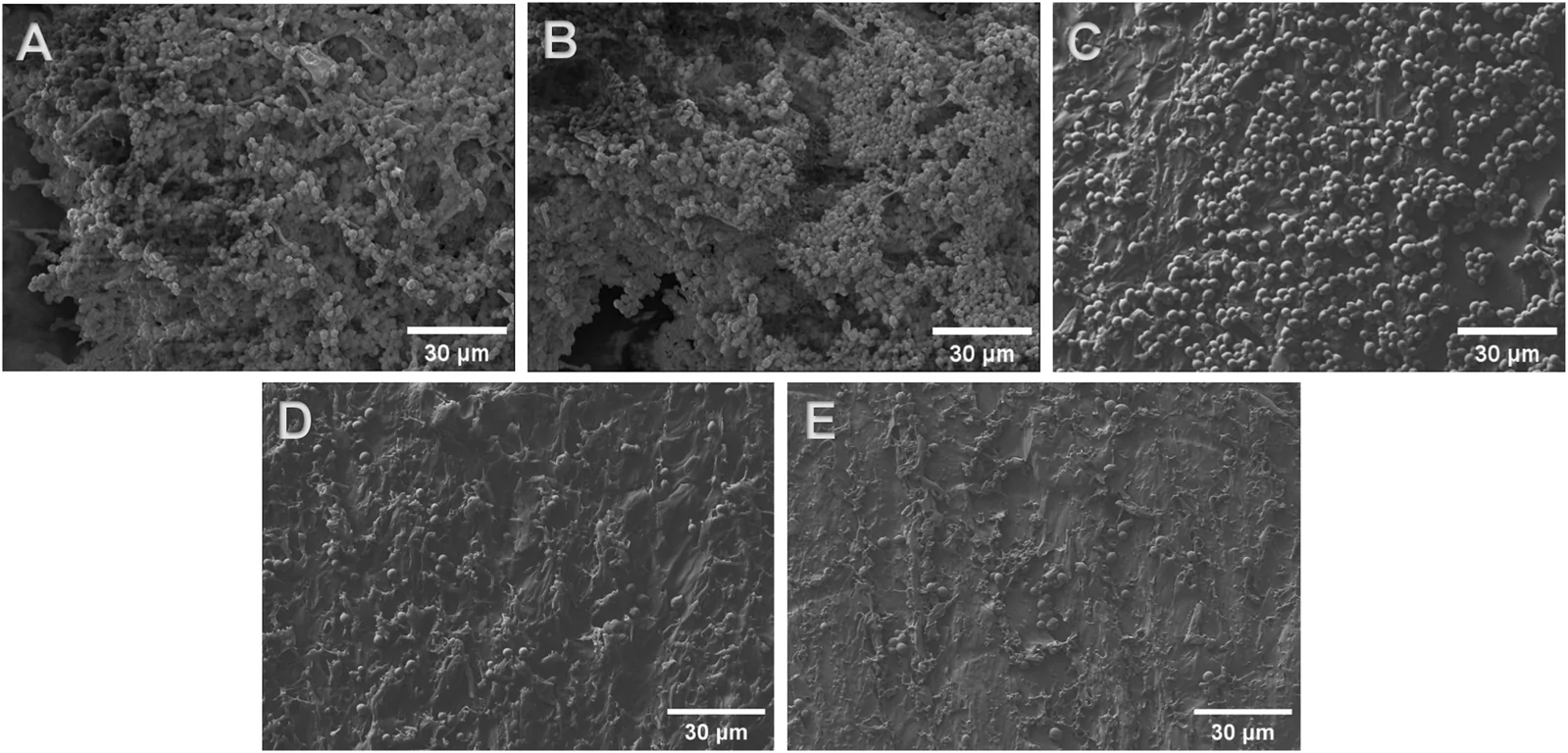
Representative scanning electron micrographs (×1,000) of *C. albicans* morphology adhered to the surface of the samples. **(A)** Biofilm on the untreated PMMA surface; **(B)** biofilm on the PMMA surface after LED light exposure (18 J/cm^2^); **(C)** biofilm on the PMMA surface after immersion in 50 μM ZnPc(Lys)_5_ for 10 min; **(D)** biofilm on the PMMA surface after immersion in 50 μM ZnPc(Lys)_5_ for 10 min followed by LED light exposure (18 J/cm^2^); **(E)** biofilm on the PMMA surface after immersion in Polident for 5 min.

In the dark toxicity group (Figure 4C), only a slight change in biofilm appearance was observed, indicating limited antifungal activity in the absence of light. In contrast, the ZnPc(Lys)_5_-mediated aPDT group (Figure 4D) and the Polident group (Figure 4E) exhibited PMMA surfaces that were markedly decreased biofilm coverage, with disrupted architecture and sparse residual cells, qualitatively consistent with the CFU reduction results.

### 3.4 Effects of ZnPc(Lys)_5_ on the microhardness and surface roughness of PMMA

To assess the impact of ZnPc(Lys)_5_ treatment on the physical properties of PMMA, surface microhardness (HV) and roughness (Ra) were evaluated across five treatment groups: 50 μM ZnPc(Lys)_5_ dark-only, no decontamination (untreated), 50 μM ZnPc(Lys)-PDT, LED-only, and Polident (Figure 3).

The HV values across all treatment groups ranged from 11.37 to 17.03, and the Ra values ranged from 0.77 to 0.95 μm. No statistically significant differences were detected among any of the five groups for either parameter (Figure 3). These findings indicate that ZnPc(Lys)_5_-mediated aPDT did not significantly alter the surface mechanical properties of PMMA, suggesting that the treatment effectively eliminates *Candida* biofilms without inducing surface softening, microcracking, or roughening of the PMMA substrate. Full statistical results are provided in Supplementary Tables S3, S4.

### 3.5 Histological evaluation in the in vivo model

#### 3.5.1 Hematoxylin and eosin (H&E) staining

To further investigate the *in vivo* efficacy of ZnPc(Lys)_5_-mediated aPDT, histological analysis was performed using a mouse oral candidiasis infection model (Figure 5).

**FIGURE 5:**
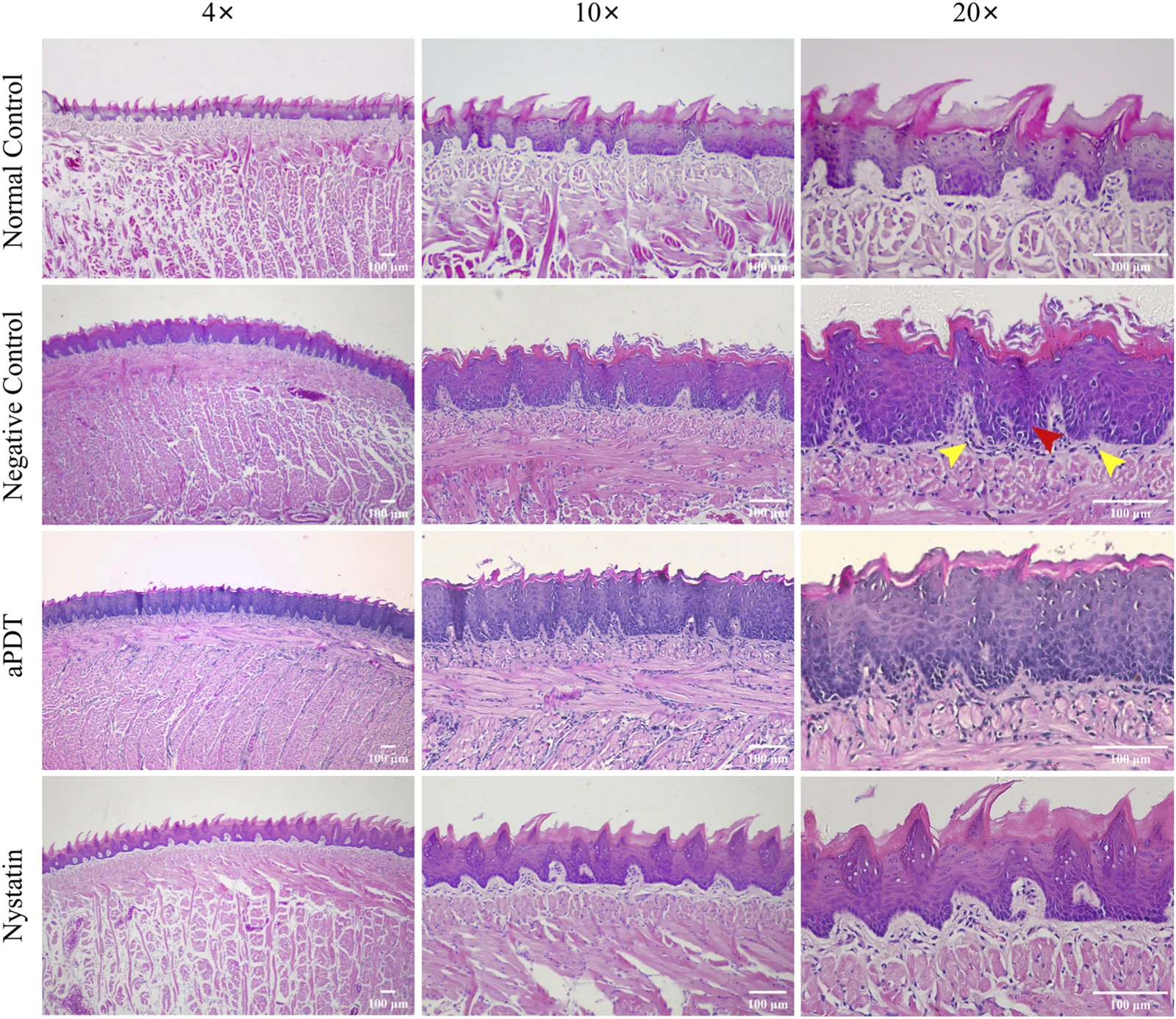
Histological staining of the dorsal tongue mucosa after treatment in each group (H&E staining, ×4, ×10, ×20). Mice were divided into three experimental groups: (1) stomatitis-negative control (infected + PBS); (2) aPDT group (infected + ZnPc(Lys)_5_ + irradiation); and (3) nystatin treatment group. An uninfected normal control group was included for histological reference. The stomatitis-negative control group shows epithelial thickening with irregular epithelial rete ridges (indicated by red arrows), and neutrophil infiltration is observed in the superficial layer of the epithelium (indicated by yellow arrows). In contrast, the aPDT and nystatin groups show reduced epithelial hyperplasia and inflammation. Scale bars = 100 μm.

The normal uninfected control group exhibited well-defined epithelial layers, orderly epithelial cell arrangement, and no evident pathological alterations. In contrast, the stomatitis-negative control group showed marked epithelial thickening, irregular epithelial rete pegs (red arrow), and superficial neutrophil infiltration (yellow arrow).

Compared with the stomatitis-negative control group, both the aPDT group and the nystatin group exhibited reduced epithelial hyperplasia and inflammatory cell infiltration, with more regular epithelial architecture. These findings indicate that ZnPc(Lys)_5_-mediated aPDT effectively alleviates the histopathological changes associated with oral candidiasis, comparable to the standard antifungal treatment.

#### 3.5.2 Periodic acid–schiff (PAS) staining

PAS staining results are presented in Figure 6. The stomatitis-negative control group showed extensive fungal colonization on the tongue surface, with numerous long and segmented hyphae penetrating the superficial epithelial layer (red arrow).

**FIGURE 6:**
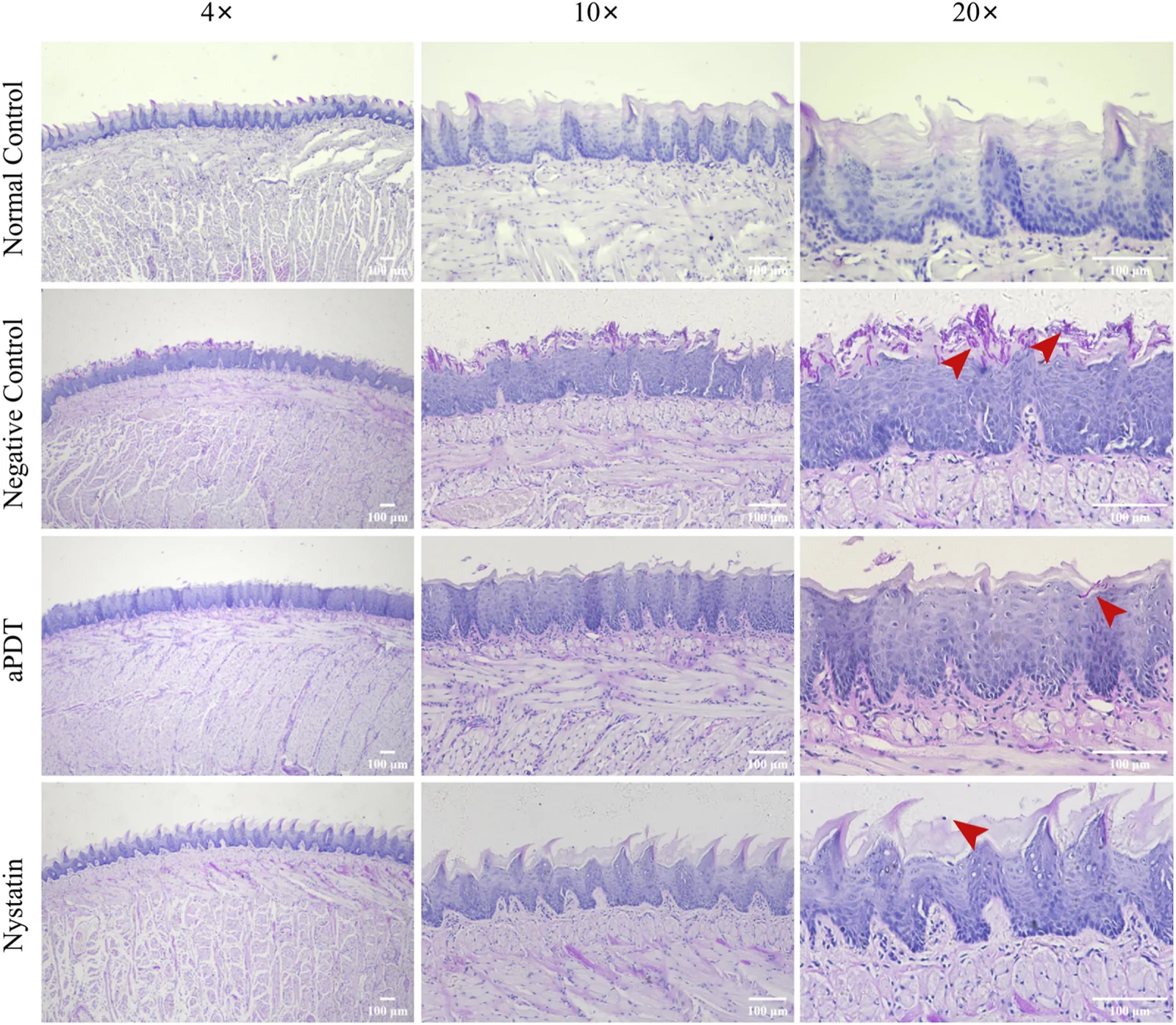
Histological staining of the dorsal tongue mucosa after treatment in each group (PAS staining, ×4, ×10, ×20). Mice were divided into three experimental groups: (1) stomatitis-negative control (infected + PBS); (2) aPDT group (infected + ZnPc(Lys)_5_ + irradiation); and (3) nystatin treatment group. An uninfected normal control group was included for histological reference. Extensive long rod-shaped or septate hyphal invasion is visible in the superficial layer of the epithelium in the stomatitis-negative control group (indicated by red arrows); whereas hyphal elements are markedly reduced in both the aPDT and nystatin groups. Scale bars = 100 μm.

In contrast, the aPDT group and the nystatin group showed significantly fewer PAS-positive fungal elements on the mucosal surface, and hyphal structures were rarely observed, appearing mainly within the keratinized layer. No obvious qualitative differences were observed between the aPDT and nystatin groups regarding the extent of fungal reduction.

Collectively, the *in vivo* data demonstrate that ZnPc(Lys)_5_-mediated aPDT effectively reduces fungal burden and improves tissue pathology in the oral candidiasis model, with an efficacy comparable to that of nystatin.

## 4 Discussion

Denture stomatitis (DS) is widely recognized as a multifactorial inflammatory condition strongly associated with *Candida* albicans biofilm formation on denture base materials (Abuhajar et al., 2023; McReynolds et al., 2023; Redding et al., 2009). The persistence of biofilms on PMMA surfaces represents a major clinical challenge due to their enhanced resistance to mechanical removal and antifungal agents (Kreve and Reis, 2025; de Freitas Fernandes et al., 2011; Wen et al., 2016). In the present study, we systematically evaluated the antifungal activity of ZnPc(Lys)_5_-mediated antimicrobial photodynamic therapy (aPDT) against planktonic *C. albicans* (Hu et al., 2018; Baltazar et al., 2015; Li et al., 2023; Dias et al., 2023), its capacity to reduce recoverable fungal cells within mature biofilms formed on PMMA surfaces (Al-Qahtani, 2022; Varela et al., 2017), its impact on PMMA surface mechanical properties (Alshehri, 2021; AlHamdan, 2023; Tan et al., 2014), and its effects on stomatitis-associated histological alterations in a mouse model of Candida-associated stomatitis (Solis and Filler, 2012; Freire et al., 2016; Okada et al., 2013).

The present study employed a polished PMMA surface as a surrogate for denture base material, simulating the non-biological substrate to which *C. albicans* adheres in clinical settings. Biofilms were cultivated in YPD medium for 48 h, a widely accepted paradigm for generating mature, structurally complex fungal biofilms *in vitro*. Accordingly, this experimental setup should be regarded as an *in vitro* proof-of-concept model designed to evaluate the decontamination efficacy of test agents against firmly adherent *C. albicans* biofilms on denture-relevant surfaces. Several inherent limitations of this model should be acknowledged: the absence of an acquired salivary pellicle, the lack of salivary flow and mechanical shear forces, and the exclusion of polymicrobial interactions present in the oral cavity. Despite these simplifications, this model provides a standardized and reproducible platform for assessing the penetration and inactivation of antifungal agents, serving as a prerequisite for future studies incorporating more physiologically complex conditions.

Our *in vitro* findings demonstrated that ZnPc(Lys)_5_-mediated aPDT exerted a significant concentration-dependent fungicidal effect on planktonic *C. albicans*. Statistical analysis revealed that the minimum effective concentration (MEC) was 25 μM, which achieved a 2.05 log_10_ reduction in viable cell counts (P = 0.0013). The fungicidal efficacy reached a plateau at concentrations of 50 μM and above, with a maximal reduction of 2.90 log_10_ observed at 200 μM. Importantly, no dark toxicity was observed across the entire tested concentration range (3.125–200 μM), confirming the light-dependent nature of the antifungal activity. Although the maximal reduction (2.90 log_10_) did not reach a frequently used 3-log_10_ benchmark in antimicrobial disinfection studies, the assay employed a single irradiation cycle and a short pre-irradiation incubation. Optimization of photosensitizer concentration, incubation time, irradiation dose, or repeated treatment cycles may further enhance antifungal efficacy. Therefore, the present results should be interpreted as demonstrating significant antifungal activity under conservative experimental parameters rather than maximal achievable disinfection performance.

Previously, Photofrin significantly inhibited the growth of *C. albicans* at a concentration of 1 μg/mL after 10 min of irradiation with white light at 15 mW/cm^2^, but its fungicidal effect was limited at concentrations below 0.3 μg/mL (Bliss et al., 2004). Furthermore, methylene blue (MB) at a concentration of 50 μM was unable to effectively reduce the number of viable *C. albicans* even after exposure to 660 nm laser light at doses of 9, 18, and 27 J/cm^2^ (Kato et al., 2013). Notably, ZnPc(Lys)_5_ in this study exhibited a fungicidal effect when irradiated with red light at 25 mW/cm^2^ for 12 min (irradiation dose of 18 J/cm^2^). Another third-generation photosensitizer, TBTCP-QY, which targets membrane aggregation-induced luminescence, completely killed *C. albicans* at a concentration of 0.8 μM after 15 min of irradiation with white light at 80 mW/cm^2^ (Wu et al., 2023). In contrast, ZnPc(Lys)_5_ in the present study achieved a substantial fungicidal effect under a relatively mild irradiation dose (18 J/cm^2^) and short incubation time, which may help limit thermal or phototoxic stress to surrounding tissues. Together, the data indicate that ZnPc(Lys)_5_ can achieve substantial photodynamic antifungal activity under relatively mild irradiation parameters in our experimental setting. Nonetheless, direct quantitative comparisons across studies should be interpreted cautiously due to differences in fungal strains, biofilm maturation conditions, irradiation intensity, wavelength, and experimental design.

Biofilm formation by *C. albicans* is a key pathogenic factor in DS. In the present study, both CFU recovery assays and SEM observations indicated marked reductions in biofilm-associated fungal cells following ZnPc(Lys)_5_-mediated aPDT. As illustrated in Figure 2, the treatment induced a significant, concentration-dependent reduction in viable biofilm cells, expressed as a percentage of reduction relative to the control. The 25 μM and 50 μM aPDT groups achieved the highest efficacy (exceeding 90% reduction), which was numerically greater than that of the 12.5 μM group, although the inter-group differences did not reach statistical significance. Notably, the antibiofilm effect appeared to plateau at 25 μM, as no significant difference was observed between the 25 μM and 50 μM groups. The 12.5 and 25 μM aPDT groups showed significantly greater reductions than their concentration-matched dark controls (P < 0.05), while the 50 μM group showed a similar trend that did not reach statistical significance (P = 0.119), confirming that the primary eradication mechanism was photodynamic inactivation rather than dark toxicity. Although complete elimination of viable biofilm-associated cells was not achieved, SEM revealed substantial disruption of biofilm architecture, with decreased surface coverage and sparse residual fungal structures, qualitatively consistent with the CFU-based reduction in viable cells, although SEM cannot quantitatively distinguish between physical detachment of biofilm material and microbial inactivation within the retained matrix. These findings suggest effective structural and functional impairment of mature biofilms under the tested conditions. *In vitro*, Polident was included as a clinically relevant denture-cleanser comparator rather than a pharmacological antifungal control. Furthermore, the *in vitro* comparison between Polident (5-min immersion) and aPDT (10-min dark incubation plus 12-min LED irradiation) represents an endpoint efficacy evaluation under each modality’s respective standard clinical protocol rather than a time-normalized kinetic comparison; artificially equalizing treatment durations would disrupt the standard operating windows of each modality.

The excellent antifungal performance of ZnPc(Lys)_5_-mediated aPDT is closely associated with its unique photophysical and interfacial properties. These properties govern a biologically responsive dynamic equilibrium between self-assembled nanostructures and photoactive monomers. In aqueous environments such as PBS, ZnPc(Lys)_5_ tends to form spherical supramolecular nano-assemblies, driven by strong π-π stacking of the planar aromatic phthalocyanine rings and peripheral hydrophobic interactions, while the positively charged lysine chains extend outward to form a hydrophilic shell, as directly visualized by TEM and SEM in prior studies (Chen et al., 2024; Jia et al., 2018; Liu et al., 2023). Spectroscopically, these aqueous aggregates exhibit a characteristic H-type stacking absorption peak around 630 nm with quenched fluorescence emission. However, upon contact with negatively charged microbial cell or biofilm interfaces, the π-π interactions holding the nano-assembly together are disrupted by the hydrophobic microenvironment of the fungal lipid bilayer, triggering the dissociation of aggregated ZnPc(Lys)_5_ into photo-active monomers, which is essential for efficient ROS generation under 680 nm illumination (Liu et al., 2018; Alhotan et al., 2021). Previous photophysical characterization revealed that ZnPc(Lys)_5_ possesses a singlet-oxygen quantum yield of 0.63 ± 0.02 under appropriate red-light excitation (Chen et al., 2010), supporting its potent photodynamic activity. Because singlet oxygen has a nanosecond-scale lifetime and a nanometer diffusion radius, the *in situ* release of monomers at the cell membrane causes localized lipid peroxidation, alters membrane permeability, and compromises biofilm integrity. When adsorbed onto microbial surfaces at micromolar concentrations relevant to this study, ZnPc(Lys)_5_ achieves a high monomer fraction up to 62.0%, which is superior to its poly-lysine substituted analogues ZnPc(Lys)_3_ and ZnPc(Lys)_7_ (Liu et al., 2018). In addition, ZnPc(Lys)_5_ shows favourable photostability against photobleaching, and its aqueous aggregates can act as a reservoir for gradual release of functional monomers, which guarantees its reliable anti-microbial outcome within our working concentration range of 12.5–50 μM (Chen et al., 2014; Alhotan et al., 2021).

Although direct *in situ* ROS quantification and fungal penetration imaging within *C. albicans* biofilm were not implemented in the current work, earlier bacterial-oriented mechanistic studies have proven that ZnPc(Lys)_5_ binds to microbial surfaces through electrostatic attraction mediated by the clustered positive charges of pentalysine residues (−ε-NH) interacting with abundant negatively charged moieties (e.g., phosphomannan, nucleic acids, and acidic polysaccharides) on the cell walls of *C. albicans* and within the extracellular polymeric substance (EPS) matrix, elevates membrane permeability after light irradiation, and evokes cellular oxidative-stress responses *via* ROS (Liu et al., 2018; Alhotan et al., 2021). The SEM results in the present study further visually confirm the destructive capacity of ZnPc(Lys)_5_-aPDT on mature *C. albicans* biofilm. Further mechanistic experiments including ROS-quencher assays and confocal observation of photosensitizer penetration into *Candida* biofilm will be pursued in future work to provide more direct evidence for this antifungal photodynamic process.

It is important to distinguish between antimicrobial inactivation within the biofilm and physical detachment of biofilm material. The CFU recovery assay employed in this study quantifies viable, culturable cells that can be detached from the PMMA surface by vortexing and subsequently form colonies on solid medium. This metric reflects the reduction in recoverable viable biofilm-associated cells rather than total biomass loss (Stepanović et al., 2000), extracellular polymeric substance (EPS) degradation, or complete physical detachment of the biofilm from the substrate. SEM analysis, while providing valuable qualitative insights into biofilm architecture and surface coverage (Heydorn et al., 2000), cannot distinguish between inactivated cells that remain adherent to the surface and cells that have been physically removed. In the context of aPDT, the primary mechanism of action is the generation of reactive oxygen species (ROS) that induce oxidative damage to microbial cellular components, leading to cell inactivation. This process may occur without concurrent physical removal of the biofilm matrix or dead cells. Therefore, the observed reduction in recoverable viable cells in this study should be interpreted as photodynamic inactivation of cells within the biofilm, rather than complete physical detachment of the biofilm structure.

Building on this distinction, future studies incorporating complementary quantitative biofilm assays—such as crystal violet staining (Stepanović et al., 2000) or confocal laser scanning microscopy with COMSTAT analysis (Heydorn et al., 2000)—would provide a more comprehensive assessment of antifungal efficacy.

Preservation of denture material integrity is essential for clinical translation (Alhotan et al., 2021; Ali et al., 2008). Because aPDT involves visible light irradiation, potential degradation of polymeric materials must be considered (Alshehri, 2021; AlHamdan, 2023). From a photochemical perspective, chemical degradation and main-chain scission of PMMA are driven by high-energy ultraviolet radiation (λ < 320 nm) (Torikai et al., 1993; Torikai et al., 1990). The 680 nm red light used for ZnPc(Lys)_5_ photoactivation carries a photon energy of approximately 1.82 eV (176 kJ/mol), which is far below the bond dissociation energy required to break PMMA covalent backbones (C–C and C–O bonds, typically >340 kJ/mol). In the present study, no significant changes in PMMA surface microhardness (HV) or roughness (Ra) were detected following ZnPc(Lys)_5_-mediated aPDT. The lack of statistically significant differences among all treatment groups, including the aPDT and Polident groups, underscores the material-preserving nature of this therapy. Furthermore, singlet oxygen (^1^O_2_) generated during aPDT has a short lifetime in aqueous systems (<200 ns) and an ultrashort diffusion distance (<20 nm); it reacts preferentially with biological electron-rich groups rather than the saturated acrylic ester backbones of PMMA (Dougherty et al., 1998). These findings support the material compatibility of ZnPc(Lys)_5_-mediated aPDT under the applied experimental conditions. Notably, these measurements were performed immediately after treatment; long-term aging, simulated brushing/chemical cycling, and repeated aPDT exposures should be evaluated in future studies, along with surface spectroscopic analyses such as FTIR and XPS, to provide direct chemical evidence of PMMA stability.

Biosafety and toxicity of ZnPc(Lys)_5_ have been extensively characterized in our previous standardized toxicological evaluations, which are directly relevant to the clinical scenario of incidental oral exposure during denture-related procedures. *In vivo* acute oral toxicity studies in mice demonstrated that a single oral gavage of ZnPc(Lys)_5_ at the maximum tested dose yielded an oral LD_0_ > 5,000 mg/kg body weight. Over a 14-day observation period, animals exhibited normal feeding, active behavior, steady weight gain, and no gross anatomical abnormalities in major organs, classifying the compound as practically non-toxic upon oral ingestion (Chen et al., 2024; Chen et al., 2016). *In vitro*, cytocompatibility evaluations across non-cancerous human cell lines, including human embryonic lung fibroblasts (HELF) and human periodontal ligament cells (hPDLCs), demonstrated negligible dark cytotoxicity (IC_0_ > 100 μM). At the working therapeutic concentration (50 μM applied topically for only 10 min), ZnPc(Lys)_5_ showed no adverse effects on human fibroblastic or epithelial cell viability. Standardized mutagenicity and irritation assessments further confirmed that ZnPc(Lys)_5_ does not induce micronucleus formation in mouse bone marrow polychromatic erythrocytes, confirming the absence of chromosomal or DNA-damaging genotoxicity in mammalian cells, and exhibits no mucosal or dermal irritation in rabbit and guinea pig models. Furthermore, confocal microscopy demonstrated that ZnPc(Lys)_5_ localizes in the cytoplasm and lysosomes of eukaryotic cells rather than the nucleus, providing an additional layer of safety by minimizing the risk of direct genomic exposure (Chen et al., 2024; Chen et al., 2016). On the basis of these well-established safety data and in adherence to the 3R principles (Replacement, Reduction, and Refinement) for animal experimentation, the *in vivo* study did not include separate photosensitizer-only (dark) or light-only control groups, as prior investigations had consistently demonstrated no antimicrobial or tissue-damaging effects of either parameter alone under the employed irradiation parameters. Collectively, these data establish a favorable biosafety profile for ZnPc(Lys)_5_, supporting its potential for clinical oral application where incidental oral exposure may occur.

In the mouse model of Candida-associated stomatitis, ZnPc(Lys)_5_-mediated aPDT effectively attenuated stomatitis-associated histological alterations, including reduced epithelial hyperplasia and inflammatory cell infiltration. The therapeutic efficacy of the aPDT group was comparable to that of the nystatin group, suggesting that ZnPc(Lys)_5_-mediated aPDT is a promising alternative for treating oral candidiasis. However, quantitative fungal burden measurements using tissue homogenate CFU assays were not performed. Consequently, the *in vivo* findings should be interpreted as histological improvements rather than definitive evidence of complete fungal clearance. Additional studies incorporating quantitative fungal load assessment would strengthen the evaluation of antifungal efficacy *in vivo*.

It is worth noting that the control regimens selected for the *in vitro* and *in vivo* experiments correspond to distinct clinical contexts. In the *in vitro* setting, the primary objective was to evaluate denture decontamination, and Polident—a widely used commercial denture cleanser—was therefore chosen as the positive control to represent the standard chemical cleaning approach in prosthodontic practice. In contrast, the *in vivo* experiment focused on mucosal candidal infection, for which nystatin, a topical antifungal agent routinely prescribed for oral candidiasis, served as the positive control. These two control strategies were not intended to be directly compared with each other; rather, each was selected to provide a clinically relevant benchmark within its respective experimental context.

Several limitations warrant consideration. The small animal sample size (n = 6 per group) may have constrained statistical robustness. Only a single *C. albicans* reference strain was tested, limiting generalizability across clinically diverse isolates. Moreover, the monospecies biofilm model does not recapitulate the polymicrobial ecology of the oral cavity. The short-term treatment and observation window further preclude conclusions regarding durability of antifungal efficacy and recurrence control. It should be noted that although the Ra values exceeded the critical threshold of 0.2 μm, above which surface irregularities may promote fungal recolonization, aPDT did not further exacerbate surface roughness compared with the control. Accordingly, the present findings should be interpreted as proof-of-concept evidence under controlled experimental conditions rather than validation of clinical efficacy in denture stomatitis.

Future studies should incorporate adequately powered *in vivo* designs, multiple clinical isolates, and polymicrobial biofilm systems that better reflect the oral microenvironment. Repeated irradiation protocols with longitudinal monitoring will be essential to determine sustained biofilm suppression and relapse dynamics. Integration of immune-competent models and quantitative fungal burden assessment will further clarify host–pathogen interactions and therapeutic durability. Such investigations are necessary to define the translational potential of ZnPc(Lys)_5_-mediated aPDT for denture-associated biofilm management.

Within the boundaries of this proof-of-concept study, ZnPc(Lys)_5_-mediated aPDT effectively reduced fungal viability in planktonic and biofilm states, disrupted biofilm architecture, and mitigated stomatitis-associated histopathological alterations *in vivo*, without compromising PMMA surface integrity. Collectively, these results support ZnPc(Lys)_5_ as a promising third-generation photosensitizer for antifungal photodynamic strategies targeting denture-associated biofilms.

The clinical relevance of these findings is further considered in the Conclusion, where we outline a dual-action strategy encompassing both *ex vivo* denture decontamination and *in vivo* mucosal treatment.

## 5 Conclusion

In conclusion, ZnPc(Lys)_5_-mediated aPDT represents a promising dual-action therapeutic strategy for clinical dental practice. From an *ex vivo* perspective, removable dentures can be chairside-soaked in a dilute ZnPc(Lys)_5_ solution for 5–10 min followed by 680 nm LED irradiation, effectively inactivating mature *Candida* biofilms within PMMA micro-porosities without inducing surface corrosion, wear, or dimensional changes—advantages over repetitive abrasive brushing or harsh chemical cleansers. From an *in vivo* perspective, for patients with active denture-induced stomatitis, topical application of ZnPc(Lys)_5_ to the affected palatal or alveolar mucosa followed by optical fiber-guided light irradiation can suppress mucosal fungal colonization and reduce inflammation, while avoiding the systemic drug interactions and microbial resistance risks associated with prolonged azole therapy. Collectively, these findings position ZnPc(Lys)_5_-aPDT as a safe, material-compatible, and clinically feasible adjunctive therapy for denture-related *Candida* biofilm management, warranting further investigation in multi-session clinical trials.

## Data Availability

The raw data supporting the conclusions of this article will be made available by the authors upon reasonable request, without undue reservation.
