# Supplementary material for: ZnPc(Lys)5-mediated photodynamic inactivation and reduction of Candida albicans biofilm-associated cells in vitro and in vivo
Source: Front Bioeng Biotechnol. 2026 Oct 7;14:1953510. doi: 10.3389/fbioe.2026.1953510 (PMC13683795; doi:10.3389/fbioe.2026.1953510)
Supplement: Supplementary file 1 [file DataSheet1.pdf]

Supplementary Table S1. Tukey's HSD multiple comparisons test results for Candida albicans viable counts (log_{10} CFU/mL) across 16 experimental groups

One-way ANOVA: $F(15, 48) = 11.13$, $P < 0.0001$. Post-hoc pairwise comparisons used Tukey's HSD test ($N = 64$, $n = 4$ per group).

Descriptive statistics

Group	n	Mean (log_{10} CFU/mL)	SD
LC (light control, 12 min)	4	7.210	0.184
Light + 3.125 µM	4	6.915	0.384
Light + 6.25 µM	4	6.675	0.573
Light + 12.5 µM	4	6.330	0.519
Light + 25 µM	4	5.490	0.980
Light + 50 µM	4	4.962	1.224
Light + 100 µM	4	4.788	1.140
Light + 200 µM	4	4.632	0.879
Dark + 3.125 µM	4	7.274	0.072
Dark + 6.25 µM	4	7.206	0.217
Dark + 12.5 µM	4	7.183	0.155
Dark + 25 µM	4	7.232	0.226
Dark + 50 µM	4	7.168	0.148
Dark + 100 µM	4	7.096	0.249
Dark + 200 µM	4	7.081	0.389
DC (untreated control)	4	7.536	0.092

Part 1. Key planned comparisons (mean difference, q, adjusted P-value, and 95% CI)

A. Light-treated aPDT groups vs untreated control (DC) — 12 min

Comparison	Mean difference (log_{10} CFU/mL)	q	Adjusted P	Sig.	95% CI of difference
LC vs DC	-0.3270	-1.098	1.0000	ns	[-1.8491, 1.1951]
aPDT 3.125 µM vs DC	-0.6220	-2.088	0.9803	ns	[-2.1441, 0.9001]
aPDT 6.25 µM vs DC	-0.8620	-2.893	0.7884	ns	[-2.3841, 0.6601]
aPDT 12.5 µM vs DC	-1.2060	-4.048	0.2702	ns	[-2.7281, 0.3161]
aPDT 25 µM vs DC	-2.0460	-6.868	0.0013	**	[-3.5681, -0.5239]
aPDT 50 µM vs DC	-2.5740	-8.640	<0.0001	***	[-4.0961, -1.0519]
aPDT 100 µM vs DC	-2.7480	-9.224	<0.0001	***	[-4.2701, -1.2259]
aPDT 200 µM vs DC	-2.9040	-9.748	<0.0001	***	[-4.4261, -1.3819]

B. Dark-toxicity groups vs untreated control (DC) — 0 min

Comparison	Mean difference (log_{10} CFU/mL)	q	Adjusted P	Sig.	95% CI of difference
Dark 3.125 µM vs DC	+0.2630	0.883	1.0000	ns	[-1.2591, 1.7851]
Dark 6.25 µM vs DC	+0.3310	1.111	1.0000	ns	[-1.1911, 1.8531]
Dark 12.5 µM vs DC	+0.3530	1.185	1.0000	ns	[-1.1691, 1.8751]
Dark 25 µM vs DC	+0.3050	1.024	1.0000	ns	[-1.2171, 1.8271]
Dark 50 µM vs DC	+0.3680	1.235	0.9999	ns	[-1.1541, 1.8901]
Dark 100 µM vs DC	+0.4400	1.477	0.9994	ns	[-1.0821, 1.9621]
Dark 200 µM vs DC	+0.4560	1.531	0.9992	ns	[-1.0661, 1.9781]

C. aPDT groups vs same-concentration dark-toxicity groups — photodynamic effect specificity

Comparison	Mean difference (log_{10} CFU/mL)	q	Adjusted P	Sig.	95% CI of difference
aPDT 3.125 vs Dark 3.125	-0.3590	-1.205	1.0000	ns	[-1.8811, 1.1631]
aPDT 6.25 vs Dark 6.25	-0.5310	-1.782	0.9956	ns	[-2.0531, 0.9911]

Comparison	Mean difference (log_{10} CFU/mL)	q	Adjusted P	Sig.	95% CI of difference
aPDT 12.5 vs Dark 12.5	-0.8530	-2.863	0.7995	ns	[-2.3751, 0.6691]
aPDT 25 vs Dark 25	-1.7420	-5.847	0.0118	*	[-3.2641, -0.2199]
aPDT 50 vs Dark 50	-2.2050	-7.402	0.0004	***	[-3.7271, -0.6829]
aPDT 100 vs Dark 100	-2.3080	-7.747	0.0002	***	[-3.8301, -0.7859]
aPDT 200 vs Dark 200	-2.4480	-8.217	0.0001	***	[-3.9701, -0.9259]

Part 2. Full pairwise comparison P-value matrices

Each matrix shows all pairwise comparisons within one time point. Shaded cells indicate statistically significant comparisons (P < 0.05).

A. 12 min — aPDT groups and untreated control (DC)

	DC	3.125 µM	6.25 µM	12.5 µM	25 µM	50 µM	100 µM	200 µM
**DC**	—	0.9803	0.7884	0.2702	0.0013 **	<0.0001 ***	<0.0001 ***	<0.0001 ***
**3.125 µM**	0.9803	—	1.0000	0.9887	0.0889	0.0026 **	0.0007 ***	0.0002 ***
**6.25 µM**	0.7884	1.0000	—	1.0000	0.2967	0.0144 *	0.0042 **	0.0013 **
**12.5 µM**	0.2702	0.9887	1.0000	—	0.8166	0.1223	0.0443 *	0.0160 *
**25 µM**	0.0013 **	0.0889	0.2967	0.8166	—	0.9959	0.9458	0.7938
**50 µM**	<0.0001 ***	0.0026 **	0.0144 *	0.1223	0.9959	—	1.0000	1.0000
**100 µM**	<0.0001 ***	0.0007 ***	0.0042 **	0.0443 *	0.9458	1.0000	—	1.0000
**200 µM**	<0.0001 ***	0.0002 ***	0.0013 **	0.0160 *	0.7938	1.0000	1.0000	—

B. 0 min — dark-toxicity groups and untreated control (DC)

	DC	3.125 µM	6.25 µM	12.5 µM	25 µM	50 µM	100 µM	200 µM
**DC**	—	1.0000	1.0000	1.0000	1.0000	0.9999	0.9994	0.9992
**3.125 µM**	1.0000	—	1.0000	1.0000	1.0000	1.0000	1.0000	1.0000
**6.25 µM**	1.0000	1.0000	—	1.0000	1.0000	1.0000	1.0000	1.0000
**12.5 µM**	1.0000	1.0000	1.0000	—	1.0000	1.0000	1.0000	1.0000
**25 µM**	1.0000	1.0000	1.0000	1.0000	—	1.0000	1.0000	1.0000
**50 µM**	0.9999	1.0000	1.0000	1.0000	1.0000	—	1.0000	1.0000
**100 µM**	0.9994	1.0000	1.0000	1.0000	1.0000	1.0000	—	1.0000
**200 µM**	0.9992	1.0000	1.0000	1.0000	1.0000	1.0000	1.0000	—

[***] P < 0.001 **[**]** P < 0.01 **[*]** P < 0.05 **[ns]** P ≥ 0.05 **[—]** diagonal (same group)

Statistical analysis: One-way ANOVA followed by Tukey's HSD multiple comparisons test across all 16 groups (N = 64, n = 4). ANOVA: $F(15, 48) = 11.13$, $P < 0.0001$.

Part 1 (Tables A–C): *Key planned comparisons with mean difference, q statistic, Tukey-adjusted P-value, and 95% CI. Negative mean differences indicate lower log_{10} CFU/mL in the first group named.*

Part 2 (Matrices A–B): *Full pairwise P-value matrices within each time point. Matrices are symmetric; shaded cells indicate P < 0.05.*

Effect size unit: Mean differences and 95% CIs are in log_{10} CFU/mL. 95% CI = mean difference ± $q_{0.05,16,48}$ × √(MS_error/4), with √(MS_error/4) = 0.2979; q = mean difference / 0.2979 (studentized range statistic).

Abbreviations: aPDT = antimicrobial photodynamic therapy; LC = light control; DC = untreated control; µM = micromolar.

Supplementary Table S2. Tukey's HSD multiple comparisons test results for Candida albicans biofilm decontamination (%)

One-way ANOVA: $F(8, 18) = 15.97$, $P < 0.0001$. Post-hoc pairwise comparisons used Tukey's HSD test. Data from 3 independent experiments (n = 3 per group).

Descriptive statistics

Group	n	Mean biofilm reduction (%)	SD
DC (negative / untreated control)	3	0.00	0.00
LC (light-only control)	3	38.44	15.50
12.5 µM (dark)	3	42.96	22.05
25 µM (dark)	3	54.00	20.72
50 µM (dark)	3	60.89	23.55
12.5 µM aPDT	3	85.45	8.08
25 µM aPDT	3	94.53	2.61
50 µM aPDT	3	96.08	2.32
Polident (positive control)	3	92.66	3.87

Part 1. Key planned comparisons (mean difference, q, adjusted P-value, and 95% CI)

A. Treatment groups vs negative control (DC)

Comparison	Mean difference (%)	q	Adjusted P	Sig.	95% CI of difference
12.5 µM aPDT vs DC	+85.450	10.457	<0.0001	***	[44.9575, 125.9425]
25 µM aPDT vs DC	+94.530	11.568	<0.0001	***	[54.0375, 135.0225]
50 µM aPDT vs DC	+96.077	11.757	<0.0001	***	[55.5842, 136.5691]
Polident (positive control) vs DC	+92.663	11.340	<0.0001	***	[52.1709, 133.1558]
12.5 µM dark toxicity vs DC	+42.960	5.257	0.0328	*	[2.4675, 83.4525]
25 µM dark toxicity vs DC	+53.997	6.608	0.0046	**	[13.5042, 94.4891]
50 µM dark toxicity vs DC	+60.887	7.451	0.0013	**	[20.3942, 101.3791]
Light toxicity vs DC	+38.443	4.704	0.0704	ns	[-2.0491, 78.9358]

B. aPDT groups vs same-concentration dark-toxicity groups (light-dependence)

Comparison	Mean difference (%)	q	Adjusted P	Sig.	95% CI of difference
12.5 µM aPDT vs 12.5 µM dark toxicity	+42.490	5.200	0.0355	*	[1.9975, 82.9825]
25 µM aPDT vs 25 µM dark toxicity	+40.533	4.960	0.0497	*	[0.0409, 81.0258]
50 µM aPDT vs 50 µM dark toxicity	+35.190	4.306	0.1187	ns	[-5.3025, 75.6825]

C. aPDT groups vs Polident positive control (efficacy equivalence)

Comparison	Mean difference (%)	q	Adjusted P	Sig.	95% CI of difference
12.5 µM aPDT vs Polident	-7.213	-0.883	0.9992	ns	[-47.7058, 33.2791]
25 µM aPDT vs Polident	+1.867	0.228	1.0000	ns	[-38.6258, 42.3591]
50 µM aPDT vs Polident	+3.413	0.418	1.0000	ns	[-37.0791, 43.9058]

D. aPDT inter-concentration comparisons (dose-response)

Comparison	Mean difference (%)	q	Adjusted P	Sig.	95% CI of difference
12.5 vs 25 µM aPDT	-9.080	-1.111	0.9959	ns	[-49.5725, 31.4125]
12.5 vs 50 µM aPDT	-10.627	-1.300	0.9887	ns	[-51.1191, 29.8658]
25 vs 50 µM aPDT	-1.547	-0.189	1.0000	ns	[-42.0391, 38.9458]

Part 2. Full pairwise comparison P-value matrix (9 groups)

All pairwise comparisons among the 9 experimental groups. Shaded cells indicate statistically significant comparisons (P < 0.05). Groups are ordered by ascending mean reduction (%).

	DC	Light	D12.5	D25	D50	A12.5	Polident	A25	A50
**DC**	—	0.0704	0.0328 *	0.0046 **	**0.0013 ****	<0.0001 ***	<0.0001 ***	<0.0001 ***	<0.0001 ***
**Light**	0.0704	—	1.0000	0.9037	0.5967	**0.0161 ***	**0.0044 ****	**0.0032 ****	**0.0024 ****
**D12.5**	0.0328 *	1.0000	—	0.9857	0.8176	**0.0355 ***	**0.0100 ***	**0.0071 ****	**0.0054 ****
**D25**	0.0046 **	0.9037	0.9857	—	0.9994	**0.2074**	**0.0679**	**0.0497 ***	**0.0381 ***
**D50**	**0.0013 ****	0.5967	0.8176	0.9994	—	**0.4874**	**0.1981**	**0.1505**	**0.1187**
**A12.5**	<0.0001 ***	**0.0161 ***	**0.0355 ***	**0.2074**	**0.4874**	—	0.9992	**0.9959**	**0.9887**
**Polident**	<0.0001 ***	**0.0044 ****	**0.0100 ***	**0.0679**	**0.1981**	0.9992	—	1.0000	1.0000
**A25**	<0.0001 ***	**0.0032 ****	**0.0071 ****	**0.0497 ***	**0.1505**	**0.9959**	1.0000	—	1.0000
**A50**	<0.0001 ***	**0.0024 ****	**0.0054 ****	**0.0381 ***	**0.1187**	**0.9887**	1.0000	1.0000	—

[***] P < 0.001 **[**]** P < 0.01 **[*]** P < 0.05 **[ns]** P ≥ 0.05 **[—]** diagonal (same group)

Statistical analysis: One-way ANOVA followed by Tukey's HSD test across all 9 groups (N = 27, n = 3 independent experiments). ANOVA: $F(8, 18) = 15.97$, $P < 0.0001$.

Outcome variable: Percentage of biofilm biomass reduction relative to DC (%). DC mean = 0 by definition.

Part 1 (Tables A–D): Key planned comparisons with mean difference, q statistic, Tukey-adjusted P-value, and 95% CI. Positive mean differences indicate greater biofilm reduction (better decontamination).

Part 2 (Matrix): *Full pairwise P-value matrix among all 9 groups. Matrix is symmetric; shaded cells indicate P < 0.05. Groups ordered by ascending mean.*

Group abbreviations: DC = negative untreated control; Light = light-only control; D12.5/D25/D50 = dark toxicity at 12.5/25/50 µM; A12.5/A25/A50 = aPDT at 12.5/25/50 µM; Polident = commercial denture cleanser (positive control).

Supplementary Table S3. Tukey's HSD multiple comparisons test results for in vivo Vickers microhardness (HV) among five treatment groups (Figure 4A)

One-way ANOVA: $F(4, 20) = 1.046$, $P = 0.4085$ (not significant). Post-hoc pairwise comparisons used Tukey's HSD test (N = 25, n = 5 per group). Homogeneity of variance confirmed by Brown–Forsythe (P = 0.8819) and Bartlett (P = 0.9459) tests.

A. Descriptive statistics

Group	n	Mean HV (kgf/mm²)	SD
50 µM $ZnPC(Lys)_5$	5	15.08	1.202
no decontamination	5	13.80	1.417
50 µM $ZnPC(Lys)_5$ -PDT	5	15.23	1.542
LED	5	13.87	1.863
Polident	5	15.05	1.605

Part 1. Key planned comparisons (mean difference, q, adjusted P-value, and 95% CI)

B. All groups vs no decontamination (untreated control)

Comparison	Mean difference	q	Adjusted P	Sig.	95% CI of difference
50 µM $ZnPC(Lys)_5$ vs no decontamination	+1.2800	1.857	0.6864	ns	[-1.6366, 4.1966]
50 µM $ZnPC(Lys)_5$ -PDT vs no decontamination	+1.4300	2.075	0.5941	ns	[-1.4866, 4.3466]
LED vs no decontamination	+0.0700	0.102	1.0000	ns	[-2.8466, 2.9866]
Polident vs no decontamination	+1.2500	1.814	0.7045	ns	[-1.6666, 4.1666]

C. Remaining inter-treatment pairwise comparisons

Comparison	Mean difference	q	Adjusted P	Sig.	95% CI of difference
50 µM $ZnPC(Lys)_5$ vs 50 µM $ZnPC(Lys)_5$ -PDT	-0.1500	- 0.218	0.9999	ns	[-3.0666, 2.7666]
50 µM $ZnPC(Lys)_5$ vs LED	+1.2100	1.756	0.7281	ns	[-1.7066, 4.1266]
50 µM $ZnPC(Lys)_5$ vs Polident	+0.0300	0.044	1.0000	ns	[-2.8866, 2.9466]
50 µM $ZnPC(Lys)_5$ -PDT vs LED	+1.3600	1.973	0.6375	ns	[-1.5566, 4.2766]
50 µM $ZnPC(Lys)_5$ -PDT vs Polident	+0.1800	0.261	0.9997	ns	[-2.7366, 3.0966]
LED vs Polident	-1.1800	- 1.712	0.7454	ns	[-4.0966, 1.7366]

Part 2. Full pairwise comparison P-value matrix (5 groups)

All pairwise comparisons among the 5 treatment groups. Shaded cells indicate statistically significant comparisons (P < 0.05); darker orange = more significant. Matrix is symmetric.

	ZnPC	No DC	PDT	LED	Polident
ZnPC	—	0.6864	0.9999	0.7281	1.0000
No DC	0.6864	—	0.5941	1.0000	0.7045
PDT	0.9999	0.5941	—	0.6375	0.9997
LED	0.7281	1.0000	0.6375	—	0.7454
Polident	1.0000	0.7045	0.9997	0.7454	—

[***] P < 0.001 [**] P < 0.01 [*] P < 0.05 [ns] P ≥ 0.05 [—] diagonal (same group)

Statistical analysis: One-way ANOVA followed by Tukey's HSD multiple comparisons test across all 5 groups (N = 25, n = 5 per group). No overall or pairwise difference reached significance (all adjusted P > 0.05); all groups therefore share the same compact letter (a) in Figure 4A. q = studentized range statistic.

Part 1 (Tables B–C): Key planned comparisons with mean difference, q statistic, Tukey-adjusted P-value, significance flag, and 95% CI.

Part 2 (Matrix): full symmetric pairwise P-value matrix.

Descriptive statistics report group means and SD. 95% CI = mean difference ± $q_{0.05,5,20}$ × √(MS_error/5), with pooled within-group MS_error = 2.375 (df = 20).

Abbreviations: ZnPC = 50 µM $ZnPC(Lys)_5$ photosensitizer only (no irradiation); no decontamination = untreated control; PDT = 50 µM $ZnPC(Lys)_5$ -mediated photodynamic therapy; LED = LED irradiation only; Polident = commercial denture cleanser (positive control); HV = Vickers microhardness (kgf/mm², test force $HV_{0.1}$; see Figure 4A legend).

Supplementary Table S4. Tukey's HSD multiple comparisons test results for in vivo surface roughness (Ra, μm) among five treatment groups (Figure 4B)

One-way ANOVA: $F(4, 20) = 0.7182$, $P = 0.5894$ (not significant). Post-hoc pairwise comparisons used Tukey's HSD test ($N = 25$, $n = 5$ per group). Homogeneity of variance confirmed by Brown–Forsythe ($P = 0.5719$) and Bartlett ($P = 0.4282$) tests.

A. Descriptive statistics

Group	n	Mean Ra (μm)	SD
50 μM $ZnPC(Lys)_5$	5	0.8300	0.0316
no decontamination	5	0.8180	0.0455
50 μM $ZnPC(Lys)_5$ -PDT	5	0.8540	0.0344
LED	5	0.8420	0.0259
Polident	5	0.8560	0.0643

Part 1. Key planned comparisons (mean difference, q, adjusted P-value, and 95% CI)

B. All groups vs no decontamination (untreated control)

Comparison	Mean difference	q	Adjusted P	Sig.	95% CI of difference
50 μM $ZnPC(Lys)_5$ vs no decontamination	+0.0120	0.631	0.9912	ns	[-0.06852, 0.09252]
50 μM $ZnPC(Lys)_5$ -PDT vs no decontamination	+0.0360	1.892	0.6718	ns	[-0.04452, 0.11652]
LED vs no decontamination	+0.0240	1.261	0.8965	ns	[-0.05652, 0.10452]
Polident vs no decontamination	+0.0380	1.997	0.6273	ns	[-0.04252, 0.11852]

C. Remaining inter-treatment pairwise comparisons

Comparison	Mean difference	q	Adjusted P	Sig.	95% CI of difference
50 μM $ZnPC(Lys)_5$ vs 50 μM $ZnPC(Lys)_5$ -PDT	-0.0240	-1.261	0.8965	ns	[-0.10452, 0.05652]
50 μM $ZnPC(Lys)_5$ vs LED	-0.0120	-0.631	0.9912	ns	[-0.09252, 0.06852]
50 μM $ZnPC(Lys)_5$ vs Polident	-0.0260	-1.367	0.8669	ns	[-0.10652, 0.05452]
50 μM $ZnPC(Lys)_5$ -PDT vs LED	+0.0120	0.631	0.9912	ns	[-0.06852, 0.09252]
50 μM $ZnPC(Lys)_5$ -PDT vs Polident	-0.0020	-0.105	1.0000	ns	[-0.08252, 0.07852]
LED vs Polident	-0.0140	-0.736	0.9843	ns	[-0.09452, 0.06652]

Part 2. Full pairwise comparison P-value matrix (5 groups)

All pairwise comparisons among the 5 treatment groups. Shaded cells indicate statistically significant comparisons ($P < 0.05$); darker orange = more significant. Matrix is symmetric.

	ZnPC	No DC	PDT	LED	Polident
**ZnPC**	—	0.9912	0.8965	0.9912	0.8669
**No DC**	0.9912	—	0.6718	0.8965	0.6273
**PDT**	0.8965	0.6718	—	0.9912	1.0000
**LED**	0.9912	0.8965	0.9912	—	0.9843
**Polident**	0.8669	0.6273	1.0000	0.9843	—

[***] $P < 0.001$ [**] $P < 0.01$ [*] $P < 0.05$ [ns] $P \geq 0.05$ [—] diagonal (same group)

Statistical analysis: One-way ANOVA followed by Tukey's HSD multiple comparisons test across all 5 groups ($N = 25$, $n = 5$ per group). No overall or pairwise difference reached significance (all adjusted $P > 0.05$); all groups therefore share the same compact letter (a) in Figure 4B. q = studentized range statistic.

Part 1 (Tables B–C): Key planned comparisons with mean difference, q statistic, Tukey-adjusted P-value, significance flag, and 95% CI.

Part 2 (Matrix): full symmetric pairwise P-value matrix.

Descriptive statistics report group means and SD; pooled within-group MS_error = 0.00181 (df = 20). 95% CI = mean difference ± $q_{0.05,5,20} \times \sqrt{MS\_error/5}$.

Abbreviations: ZnPC = 50 μM $ZnPC(Lys)_5$ photosensitizer only (no irradiation); no decontamination = untreated control; PDT = 50 μM $ZnPC(Lys)_5$ -mediated photodynamic therapy; LED = LED irradiation only; Polident = commercial denture cleanser (positive control); Ra = arithmetic mean surface roughness in μm (see Figure 4B legend).

Supplementary Table S5. One-way ANOVA summary statistics

Assay	Groups (k)	df (between, within)	F	P	η^2
Planktonic CFU (Fig. 1)	16	15, 48	11.13	<0.0001	0.777
Biofilm CFU (Fig. 2)	9	8, 18	15.97	<0.0001	0.877
PMMA microhardness (Fig. 4A)	5	4, 20	1.05	0.405	0.174
PMMA roughness (Fig. 4B)	5	4, 20	0.72	0.590	0.126

Note: η^2 = eta-squared (effect size). All assays used one-way ANOVA followed by Tukey HSD post-hoc tests. Complete pairwise comparison results are provided in Supplementary Tables 1–4.
